# Fibrinogen and albumin synthesis rates in major upper abdominal surgery

**DOI:** 10.1371/journal.pone.0276775

**Published:** 2022-10-27

**Authors:** Gabriel Dumitrescu, Anna Januszkiewicz, Anna Ågren, Maria Magnusson, Ernesto Sparrelid, Olav Rooyackers, Jan Wernerman

**Affiliations:** 1 Division of Anaesthesia, Department of Clinical Science, Intervention and Technology, Karolinska Institutet, Stockholm, Sweden; 2 Department of Molecular Medicine and Surgery, MMK, Clinical Chemistry and Coagulation, Karolinska Institutet, Stockholm, Sweden; 3 Division of Paediatrics, Department of Clinical Science, Intervention and Technology, Karolinska Institutet, Stockholm, Sweden; 4 Division of Surgery, Department of Clinical Science, Intervention and Technology, Karolinska Institutet, Stockholm, Sweden; Institute of Experimental Hematology and Transfusion Medicine, University Clinic of Bonn, GERMANY

## Abstract

Plasma fibrinogen and albumin concentrations initially decrease after abdominal surgery. On postoperative days 3–5 fibrinogen concentration returns to the preoperative level or even higher, while albumin stays low. It is not known if these altered plasma concentrations reflect changes in synthesis rate, utilization, or both. In particular a low albumin plasma concentration has often been attributed to a low synthesis rate, which is not always the case. The objective of this study was to determine fibrinogen and albumin quantitative synthesis rates in patients undergoing major upper abdominal surgery with and without intact liver size. Patients undergoing liver or pancreatic resection (n = 9+6) were studied preoperatively, on postoperative days 1 and 3–5. De novo synthesis of fibrinogen and albumin was determined; in addition, several biomarkers indicative of fibrinogen utilization were monitored. After hemihepatectomy, fibrinogen synthesis was 2-3-fold higher on postoperative day 1 than preoperatively. On postoperative days 3–5 the synthesis level was still higher than preoperatively. Following major liver resections albumin synthesis was not altered postoperatively compared to preoperative values. After pancreatic resection, on postoperative day 1 fibrinogen synthesis was 5-6-fold higher than preoperatively and albumin synthesis 1.5-fold higher. On postoperative days 3–5, synthesis levels returned to preoperative levels. Despite decreases in plasma concentrations, de novo synthesis of fibrinogen was markedly stimulated on postoperative day 1 after both hemihepatectomies and pancreatectomies, while de novo albumin synthesis remained grossly unchanged. The less pronounced changes seen following hepatectomies were possibly related to the loss of liver tissue.

## Introduction

The alterations in plasma concentrations of important hepatic export protein are well characterized in conjunction with major surgical traumas [1–5]. The relationships between changes in plasma concentrations and de novo synthesis and utilization are however not. In a recent exploratory study of patients undergoing hepatic surgery, focusing on coagulation, we found that restitution of the reduction of the plasma fibrinogen concentration seen on the first postoperative day, is related to the size of the resection [1]. Patients undergoing hemihepatectomy exhibit an overshoot in plasma concentration on the fourth postoperative day, whereas patients undergoing extended hemihepatectomy do not. This postoperative pattern is also in agreement with other reports [3]. Therefore, understanding the genesis and significance of changes in plasma concentrations of liver export proteins, particularly when surgery involves a reduction of liver tissue mass may be crucial.

Major liver resections have become increasingly safe due to improvements in patient selection and surgical techniques. Still, liver surgery is associated with a risk for postoperative liver insufficiency that is proportional to the resection size and quality of the remnant liver [6,7].

Computer tomography volumetric analysis or magnetic resonance imaging alone or combined with hepatobiliary scintigraphy are used to estimate the functional reserve of the liver and indirectly to assess the risk of postoperative liver failure [8–12]. However, the post-resection functional status of the liver, particularly the synthetic function for export proteins of the remnant liver has not been explored.

To explore the mechanisms behind the difference in postoperative plasma concentration of fibrinogen and albumin related to hepatic resection, we designed a study to assess the de novo synthesis rates of two liver export proteins fibrinogen and albumin in patients undergoing liver resections of varioussizes. Our hypothesis was that the postoperative fibrinogen and albumin plasma levels are related to their de novo synthesis. Patients undergoing pancreatic resections- with intact liver size served as controls. Coagulation activation tests to explore the fibrinogen catabolic pathways were added. Thromboelastometry was used to explore coagulability which is affected by fibrinogen [1,13,14].

## Materials and methods

### Subjects

Patients with an indication for hepatectomy or pancreatic resection at the Karolinska Huddinge University Hospital in Stockholm were recruited between September 2017 and May 2019. Smokers and patients with coagulopathies’ or who were undergoing treatment with anticoagulants or platelets aggregation inhibitors were excluded. The patients gave their written consent after being informed about the study protocol orally and in writing. The protocol was approved by the Regional Ethics Committee in Stockholm and was in conformity with the Declaration of Helsinki of 1975. The study was registered at ANZCTR, ID ACTRN12617000749303.

In total 15 patients were enrolled in two groups: 1) large liver resections (n = 9) and 2) pancreas resections (n = 6). The patients’ characteristics, diagnosis, and surgical interventions are given in Table 1.

**Table 1:** Patients’ characteristics, diagnosis and surgical interventions.

Patients	Liver surgery	Pancreas surgery
**No.**	9	6
**Sex** Male Female	5 4	1 5
**Age** (years)^a^	59(43–77)	70(60–78)
**Height** (cm)^a^	1.73 (1.59–1.85)	1.67(1.61–1.71)
**Weight** (kg)^a^	69(43–85)	77(61–95)
**Diagnosis** Colorectal metastasis IPMN^b^ Pancreas cystic tumor	9 - -	- 5 1
**Performed surgery** Resection of two liver segments and right colectomy Right hemihepatectomy Extended right hemihepatectomy PVE with two-stages hepatectomy^c^ Distal pancreatectomy Total pancreatectomy	1 5 1 2 - -	- - - - 4 2
**Postoperative thrombosis** ^d^	0	1

^a^Median (range).

^b^IPMN = Intraductal papillary mucinous neoplasm.

^c^portal vein embolization/ligation (PVE) with two-stages right hemihepatectomy.

^d^portal vein thrombosis.

The size of the samples (N = 9 + 6) was based on a power analysis (2-sided test) that assumed a difference of 1.5 standard deviation between the two groups, normal distribution, and alpha < .05 for direct comparisons, which give a power of 0.8. For the power analysis Statistica 13 was used.

Studies exploring fibrinogen synthesis in humans are rare, and in patients undergoing surgery nonexistent to our knowledge. Based on previously published studies on liver insufficiency caused by cirrhosis [15] and pancreas cancer with an acute phase response [16], our best-educated guess is that an effect size of the studied major abdominal surgery on fibrinogen synthesis of at least 1.5 would be appropriate to be considered in the power analysis to detect relevant changes in synthesis rates.

### Protocol

De novo synthesis rates of fibrinogen and albumin were studied in a longitudinal protocol at three times: preoperatively and on postoperative day 1 and postoperative days 3–5. The patients who underwent portal vein embolization with two-stage hemi-hepatectomy were studied preoperatively and after the second stage of the operation (postoperative days 1 and 3–5). At the same time, blood samples for coagulation and biochemical tests were collected.

In a pre-study employing healthy volunteers we concluded that a maximum of 3 consecutive samples in the same subjects not less than 24 hours apart and using the same isotopic label gave the best precision in determining the synthesis rate in multiple samples [17].

All procedures in conjunction with anesthesia, surgery, and postoperative care were in accord with the local routines followed at the Karolinska Huddinge University Hospital. The general anesthesia was performed with propofol-fentanyl induction and maintenance with sevoflurane-fentanyl. Epidural analgesia was administrated intraoperatively and postoperatively for 5–7 days. Intraoperatively fluids were administered intravenously in the form of glucose 2.5% 1 ml/kg/hour and Ringer acetate 4 ml/kg/hour.

All patients received daily thrombosis prophylaxis with 5000 IU dalteparin (Fragmin®) at 8:00 p.m. under the entire hospitalization, starting on the day before the operation.

Preoperatively the patients were investigated in a post-absorptive state (more than 6 hours after a warm meal, and more than 4 hours after coffee or tea. Tap water was allowed without restriction). Postoperatively enteral nutrition was initiated as soon as possible and was stopped 6 hours before measurement. Intravenous glucose solution, when given (at a rate of 0.5 -1ml/kg/hour), was paused not less than 2 hours before measurement. The intravenous crystalloid solution Ringer acetate 1–2 ml/kg/hour was administered postoperatively.

#### Analytic methods for de novo protein synthesis

We used the flooding dose technique with ^2^H_5_-phenylalanine for the determination of the synthesis rates of fibrinogen and albumin which was described previously [17,18].

In short, a large dose of labelled phenylalanine was given as a bolus to flood all compartments and thus make the labelling in the immediate precursor pool for protein synthesis in the hepatocytes similar to that in plasma.

After precipitation and elimination of plasma proteins, ^2^H_5_- and unlabeled phenylalanine in plasma were measured as their tert-butyldimethylsilyl derivative using gas chromatography-mass- spectrometry (Agilent 5975C, Agilent, Kista, Sweden).

For the enrichment measurements of albumin and fibrinogen, these two proteins were isolated from plasma and thereafter hydrolysed. Following hydroxylation, the enrichment of phenylalanine from both proteins was analyzed after conversion of phenylalanine, which makes it possible to extract it from other amino acids and to eliminate the background noise to almost zero to optimize the signal-to-noise ratio, as the labelled fraction in the incorporated protein was much lower compared to plasma. The gas chromatography-mass spectrometry analysis was then technically similar to that for plasma phenylalanins.

#### Calculation of fractional and absolute synthesis rates

Fractional synthesis rates (FSR), representing the ratio (as a percentage) of the intravascular protein mass synthesized per 24 hours, were calculated by dividing the increase of enrichment of the ^2^H_5_-phenylalanine incorporated in the protein (fibrinogen or albumin) to the area under the curve for ^2^H_5_-phenylalanine in its free pool (adjusted for the secretion time of the protein) and multiplied by 100 [17,18].

The absolute synthesis rates (ASR), representing the total amount of protein synthesized per day (mg/kg/day), were calculated by multiplying corresponding FSR with the total plasma protein (fibrinogen or albumin) mass, divided by the bodyweight [17]. Plasma volume was estimated using a dedicated formula based on anthropometric data [19].

#### Laboratory analysis

On Sysmex Sysmex CS-5100 (Sysmex Corporation, Kobe, Japan): prothrombin time-international normalized ratio (Medirox Owren´s PT reagent), activated partial thromboplastin time (aPTT) (Dade Actin FS activated PTT reagent, Siemens), fibrinogen (Clauss method, Dade Thrombin Reagent, Siemens), D-dimer (Innovance D-Dimer, Siemens), soluble fibrin monomers (Sta-Liatest FM, Stago). Platelet count was performed on Sysmex XN (Sysmex Corporation, Kobe, Japan). Plasma albumin was performed on Cobas 8000 c701 (Roche Diagnostics) with BCP (Bromocresol Purple) reagent (Roche Diagnostics).

On a SpectraMax® i3x Multi-Mode microplate reader: thrombin-antithrombin complexes (Enzygnost TAT micro Kit, Siemens) and fibrinopeptide B (Human Fibrinopeptide B Elisa Kit, Novus Biologicals).

Thromboelastometry was performed using a ROTEM® delta device (Pentapharm GmbH, Munich, Germany).

#### Statistics

Normality was tested with Shapiro-Wilk test. The repeated measures analysis of variances within groups was done using one-way ANOVA, or mixed-effects model-analysis (in case of missing values), and between groups using mixed-effects model analysis. Tukey’s method was used for multiple comparisons. Pearson’s correlation test was used for normally distributed variables and Spearman’s correlation test for non-normally distributed variables. The level for acceptable statistical significance (p) was < 0.05.

For the statistical analysis GraphPad Prism 8 (GraphPad Software Inc, La Jolla, CA) was used.

## Results

### Plasma concentrations of fibrinogen and albumin

**Fig 1:**
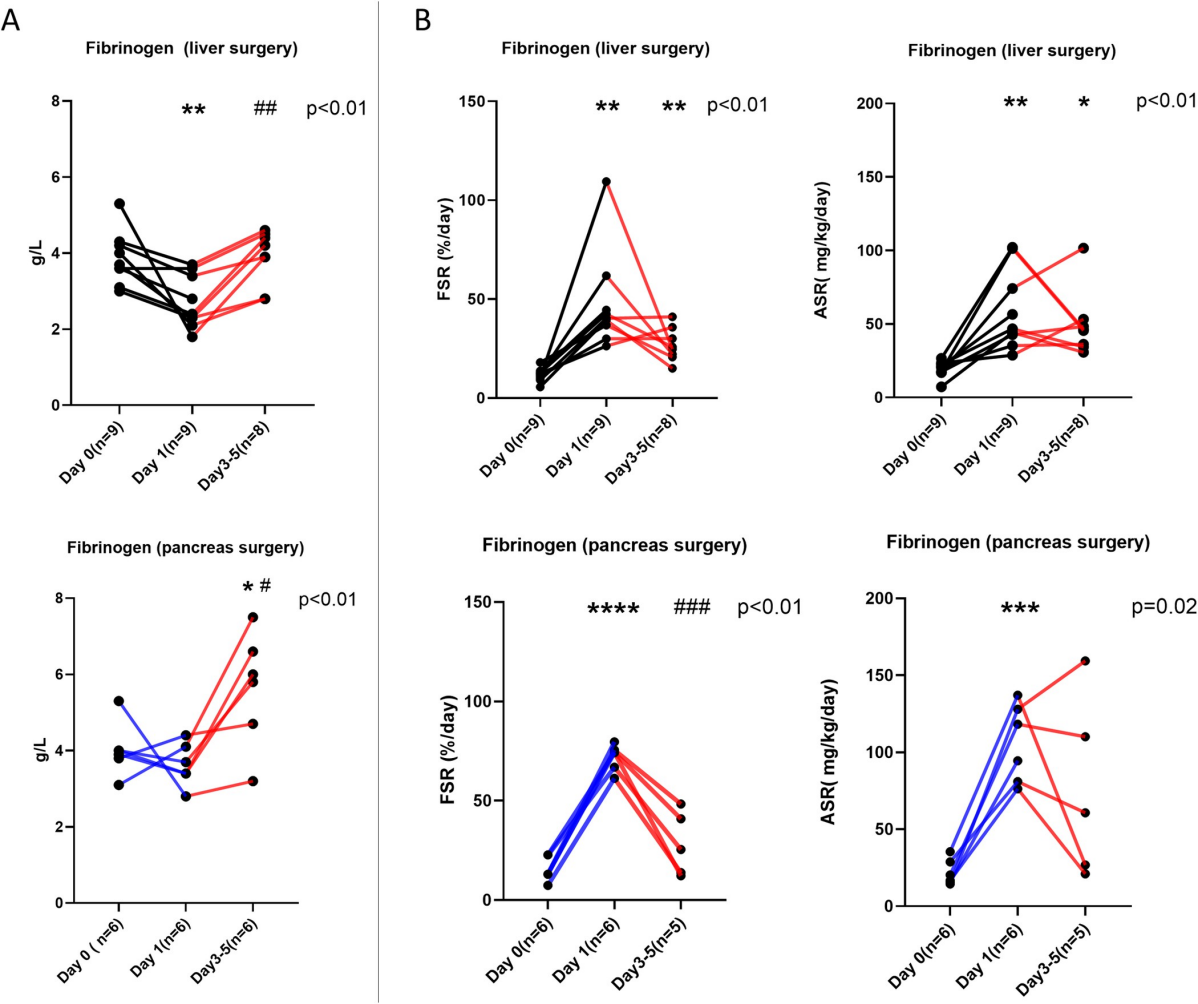
The fibrinogen plasma concentrations (A) and synthesis rates (B). The individual values for all patients undergoing liver surgery and pancreas surgery are depicted as black bold points. Levels of statistical significance for the ANOVA (or mixed-effects model analysis within groups) are given in the upper right corner. For multiple comparisons *, **, ***, **** denotes the statistical significance p < 0.05, p < 0.01, p<0.001 and < 0.0001 respectively of the difference from preoperative values, while # have the same significance from postoperative day 1.

Plasma concentrations of fibrinogen and albumin are given in Figs 1 (panel A) and 2 (panel A), respectively. The two groups exhibited different patterns. For fibrinogen, following hepatectomy, a decrease on postoperative day 1 was followed by restitution to preoperative values on days 3–5. By contrast, the patients who underwent pancreatectomy had unaltered concentrations of fibrinogen on postoperative day 1 followed by an overshoot on postoperative days 3–5. For albumin, there was a decrease in plasma concentration for both groups on postoperative day 1, but larger for the pancreatectomy group, which remained low compared to preoperative values on postoperative days 3–5.

**Fig 2:**
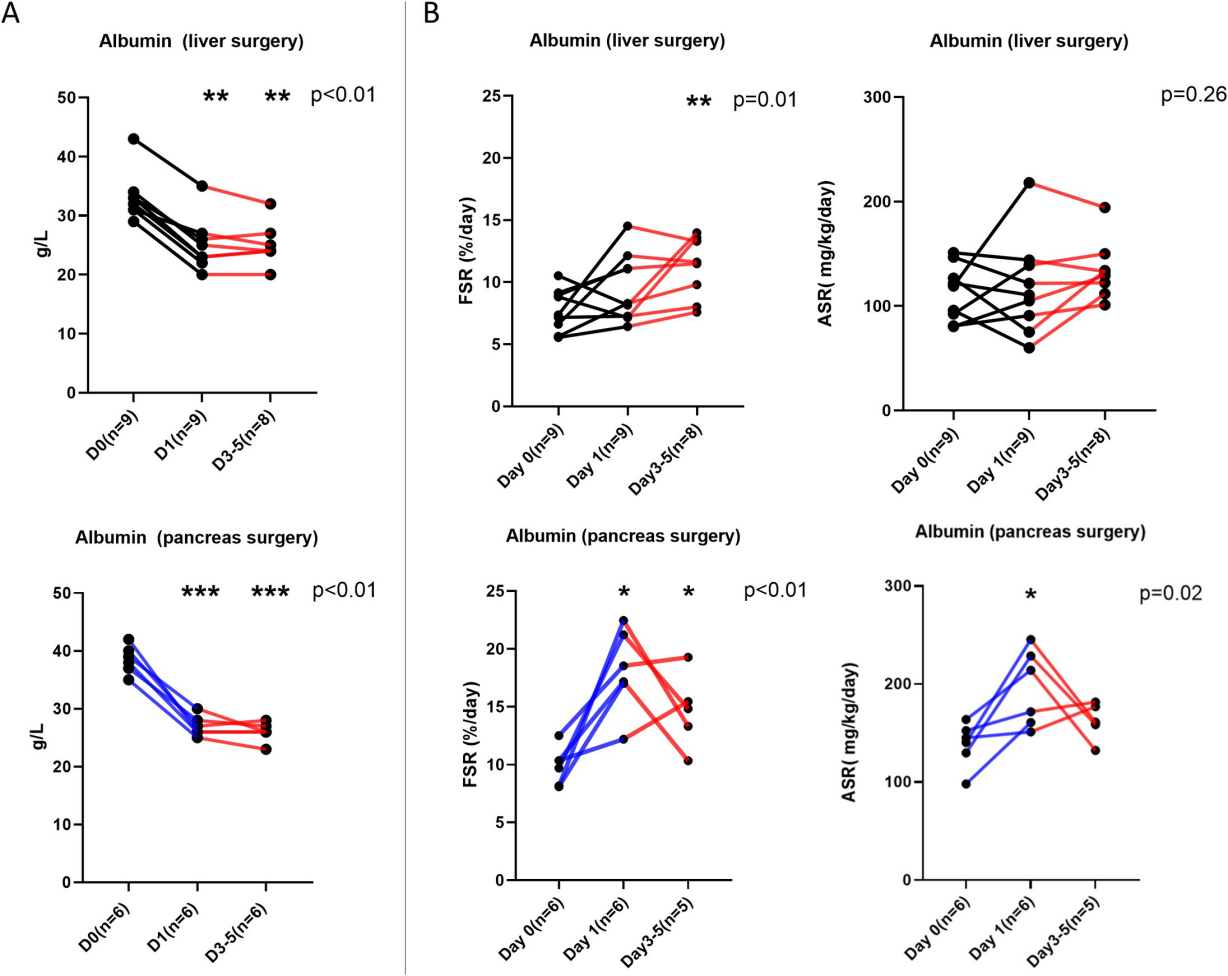
The albumin plasma concentrations (A) and synthesis rates (B). The individual values for all patients undergoing liver surgery and pancreas surgery are depicted as black bold points. Levels of statistical significance for the ANOVA (or mixed-effects model analysis within groups) are given in the upper right corner. For multiple comparisons *, **, ***, **** denotes the statistical significance p < 0.05, p < 0.01, p<0.001 and < 0.0001 respectively of the difference from preoperative values, while # have the same significance from postoperative day 1.

**Fig 3:**
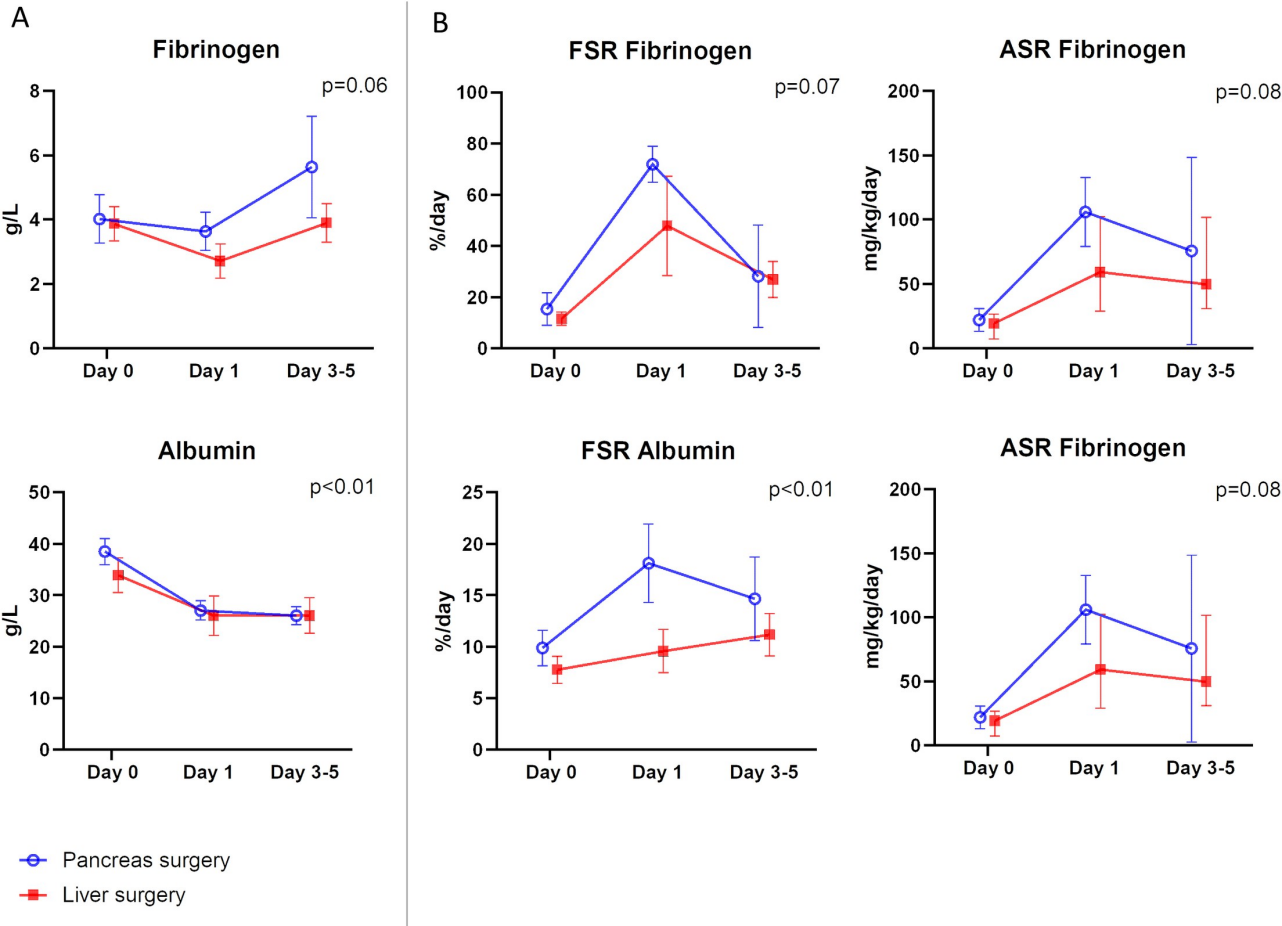
The temporal pattern of fibrinogen and albumin plasma concentrations (A) and synthesis rates (B). Liver surgery is depicted in red and pancreas surgery in blue. Values are provided as the mean + 0.95 confidence intervals and the level of statistical significance for mixed-effects model analysis between groups is given in the upper right corner.

Mixed-effects model analyses between the two groups are given in Fig 3 (panel A). The differences were not statistically significant for trends of fibrinogen plasma concentrations. Differences in albumin plasma concentration trends between the two groups were attributed to the lower preoperative values in patients undergoing liver surgery.

### De novo synthesis of fibrinogen and albumin

Results for fibrinogen synthesis are given in Fig 1 (panel B). The fractional synthesis rates were markedly increased on postoperative day 1 for both groups of patients, followed by a relative lowering of values on postoperative days 3–5. The interindividual scatter was larger, so the values on postoperative days 3–5 remained more marginally elevated for hepatectomies but were not different from the preoperative ones for pancreatectomies. When the absolute synthesis rate values were calculated, they followed the same pattern.

Albumin synthesis rates are given in Fig 2 (panel B). For the hepatectomy group the synthesis rates were unaltered on postoperative day 1, but the fractional synthesis rate increased on postoperative days 3–5. The pancreatectomy group showed an increase in both the fractional and absolute albumin synthesis rate on postoperative day 1, which remained higher for the fractional synthesis rate, but not for the absolute synthesis rate on postoperative days 3–5.

Mixed-effects model analyses between the two groups are given in Fig 3 (panel B). There were no statistically significant differences between pancreas and liver surgery regarding the synthesis of fibrinogen, but for albumin the synthesis rate trends were different.

### Biochemical and coagulation analyses

Laboratory analyses are given in Table 2. The bilirubin plasma concentrations were increased following liver surgery during the entire observed postoperative period, while in pancreas surgery they were unchanged. For PT-INR there were increased values both for hepatectomy and pancreatectomy patients especially on postoperative day 1. APTT and platelet count were not altered postoperatively regardless of the type of operation.

**Table 2:** Laboratory tests.

	Ref. range		Day 0	Day 1	Day 3–5	p_0_	p
**Bilirubin**	< 26 μmol/ml	L	**10**(5–17)	**21**(9–52) *	**20**(5–44) *	0.02	0.10
		P	**6**(4–9)	**9**(4–15)	**6**(4–15)	0.3	
**Albumin**	34–45 g/L	L	**33**(29–43)	**25**(20–35) **	**25**(20–32) **	<0.01	<0.01
		P	**38**(35–42)	**26**(25–30) ***	**26**(23–28) ***	<0.01	
**Fibrinogen**	2–4.2 g/L	L	**3.7**(3–5.3)	**2.4**(1.8–3.7) **	**4**(2.8–4.6) ##	<0.01	0.06
		P	**3.9**(3.1–5.3)	**3.5**(2.8–4.4)	**5.9**(3.2–7.5) *#	<0.01	
**PT-INR**	<1,2	L	**1.0**(0.9–1.1)	**1.5**(1.4–1.9) ***	**1.3**(1.1–1.5) *#	<0.01	0.04
		P	**0.9**(0.9–1.2)	**1.2**(1.1–1.6) **	**1.2**(1–1.4) **	<0.01	
**APT**	28–40 s	L	**23**(22–29)	**25**(24–32)	**25**(22–32)	0.1	0.7
		P	**23**(22–26)	**25**(22–28)	**25**(21–34)	0.5	
**Platelets count**	165–387 x10(9)/L	L	**180**(149–358)	**177**(112–214)	**167**(138–250)	0.25	0.7
		P	**233**(188–322)	**168**(154–305) *	**202**(111–441)	0.29	
**Thrombin-antithrombin complexes(TAT)**	8.8 ± 1.8 pg/ml	P	**5.8**(3–11.3)	**27.7**(11.9–77.3) *	**6.9**(6.4–27) #	0.01	0.95
		L	**9.7**(2.4–57)	**36**(11–76) *	**15.4**(5.2–32.4)	0.03	
**Soluble fibrin**	< 9 mg/L	L	**5.0** (1–7)	**51** (5–150) **	**8**(2–150)	0.01	0.03
		P	**3.5** (1–5)	**6** (5–17)	**5.5** (2–12)	0.07	
**Fibrinopeptide B(fpB)**	μg/ml (no ref. range available)	L	**44**(31–64)	**50**(50–78)	**45** (32–64)	0.57	0.44
		P	**44**(30–60)	**42**(39–53)	**38**(28–41)	0.29	
**D-dimer**	<0.7mg/L	L	**0.8**(0.4–1.7)	**6.1**(0.9–19.2) **	**8.2**(4.7–14.8) **	<0.01	0.07
		P	**0.6**(0.2–1.7)	**3.3**(1.9–6.4) *	**4.0**(1.5–6.7) *	0.02	

Laboratory analyses in patients undergoing liver surgery (n = 9) and pancreas surgery (n = 6) given as median (range). Levels of statistical significance (p0) for the ANOVA (or mixed-effects analysis within groups) and (p) for mixed-effects model analysis between groups are given. (L = liver surgery, P = pancreas surgery). For multiple comparisons *, **, ***, **** denotes the statistical significance p < 0.05, p < 0.01, p<0.001 and < 0.0001, respectively, of the difference from preoperative values, while # have the same significance from postoperative day 1.

The S1 Fig contains a graphic representation of data for coagulation activation markers thrombin-antithrombin complexes (TAT), soluble fibrin, fibrinopeptide B (fpB) and D-dimer. Following both liver and pancreas surgery, TAT levels were higher on postoperative day 1 than preoperatively, decreasing towards normalization on postoperative days 3–5. Soluble fibrin concentrations were increased on postoperative day 1 only after liver surgery, while fpB levels were unaltered in both groups regardless of what postoperative day. D-dimer concentrations increased postoperatively, and that was more accentuated following liver surgery. There were no statistically significant differences between liver and pancreas surgery regarding the coagulation markers except for soluble fibrin.

#### The results from the thromboelastometry

The thromboelastometric results are given in the figures included in the S2 Fig (panel A). The analyzed thromboelastometric parameters for INTEM and EXTEM were the clotting time (CT), representing the time in seconds from the start of the analysis to the initiation of clotting; clot formation time (CFT), representing the time in seconds from the initiation of clotting until an amplitude of 20 mm of the graphic trace was reached; and maximum clot firmness (MCF), representing the maximal amplitude (mm) of the graphic trace of clot firmness. For FIBTEM only MCF was investigated. For both groups, the mean values of most thromboelastometric parameters remained within the normal range during the entire study period. The exception was the MCF-FIBTEM values which tended to exceed the hypercoagulability on POD 3–5. There were no statistically significant differences in temporal pattern between the two groups.

The small temporal changes regarding CFT and MCF were parallel with the fibrinogen plasma concentrations and not with the fibrinogen synthesis rates, which moved in opposite directions (see S2 Fig—panel B).

#### Hypercoagulability signs

On POD 3–5 most patients who underwent a pancreatectomy had plasma fibrinogen concentrations, as well as MCF-FIBTEM, over the normal range **(**S2 Fig).

In two patients who underwent distal pancreatectomies, on POD 4 and 5, respectively, the ROTEM parameter MCF increased over the normal in all three curves INTEM, EXTEM and FIBTEM, and fibrinogen plasma level was over 6 g/L. In one of these patients, a routine CT scan on postoperative day 8 disclosed a fresh portal vein thrombosis.

## Discussions

The focus of the present study was to explore how plasma concentrations and de novo synthesis of two major hepatic export proteins were affected perioperatively by major abdominal surgery with and without a reduction in liver mass.

Following both major liver resections and pancreatic resections there was a dramatic increase in the de novo synthesis rate of fibrinogen on postoperative day 1, and this was most pronounced in the pancreatectomy group with an intact liver mass. As far as we are aware, this physiological response has not been described before. It is known that interleukin 6, the main inducer of fibrinogen synthesis exhibits a peak in plasma concentration at 12–24 hours postoperatively [20], similar to the fibrinogen synthesis trends described in our study. Postoperative levels (or changes in levels) of plasma fibrinogen concentrations were not indicators of levels (or changes in levels) of synthesis. On the contrary, plasma concentrations were marginally decreased (in all subjects who underwent a hepatectomy) or unaltered (pancreatectomy) on postoperative day 1, which was in agreement with previous studies [1,3,21]. At this time, the plasma fibrinogen concentrations were likely on an upward trend, as their nadir, according to previous studies, occurs at the end of the operation [3,22]. On postoperative days 3–5 this increase in de novo synthesis levelled off, but the plasma concentration revealed an overshoot compared to preoperative values in the pancreatectomy patients with the intact liver mass. Although heparin administration over a long period of time is reported to decrease plasma fibrinogen [23], it is unlikely that the low doses of low molecular weight heparin administered to our patients affected the levels or trends of plasma concentrations of fibrinogen [24]. In parallel the indirect biomarkers of fibrinogen utilization showed an activation on postoperative day 1 (TAT and soluble fibrin), and a more prolonged activation (D-dimer), in which the difference between the hepatectomy and pancreatectomy groups was equivocal. For coagulation competence, the global measures via tromboelastometry showed balanced coagulation for both groups of patients.

The study design does not allow for any conclusion over why there were lower concentrations of plasma fibrinogen on postoperative day 1 despite higher synthesis rates. One may speculate over an enhanced use of substantial amounts of de novo synthesized fibrinogen, which may be estimated to be on average 2 g/day for hepatectomies, and 6 g/day for pancreatectomies. Taking into consideration the fibrinogen half-life, which under normal conditions is around 4 days [25], this additional output of fibrinogen should have been noticed in plasma concentrations unless a fast utilization occurred. The fibrinogen exit from the plasma pool is commonly associated with fibrin formation. The utilization of fibrinogen in the coagulation process is known to be increased intraoperatively compared to normal conditions [26]. In our study, TAT levels on postoperative day 1 may be interpreted to indicate a certain coagulation activation and, implicitly, an increased fibrinogen utilization, to the same extent in liver and pancreas surgery. However, the levels of soluble fibrin monomers showed that fibrinogen consumption by fibrin formation, which is exacerbated intra-operatively [27,28], decreased hastily after pancreatectomies, while following major liver surgery the process was ongoing at higher levels even on postoperative day 1. The magnitude of the active fibrin formation, as indicated by fpB levels, seemed not to be high on postoperative day 1 and onwards.

There is evidence that even under trauma or surgical operations fibrin formation might not be the main catabolic pathway for fibrinogen [26]. An increased trans-capillary escape could also be involved [25]. Unfortunately, although intuited, this pathway is not described until now [29,30]. Our study thus opens a new perspective towards the determination of fibrinogen extravasation. A methodology to study the fibrinogen escape rate from the intravascular space is possible. The technology using metabolic isotope tracers allows to determine the in vivo kinetics of molecules [31] and was used by our group in previous studies on albumin [2]. This research would complete the lack of knowledge necessary to understand the mechanism which governs the fibrinogen plasma concentrations following major abdominal surgery with or without loss of liver mass.

Plasma fibrinogen is essential for balanced coagulation. ROTEM showed mainly balanced coagulation following both types of surgery, which for liver surgery is in agreement with previous studies [1,22]. Following pancreatectomies, increased plasma fibrinogen concentrations on postoperative days 3–5 affected the ROTEM firmness parameter MCF, pushing it towards the hypercoagulability area. The portal vein thrombosis observed in one of the patients with signs of hypercoagulability in three ROTEM parameters could be related to a pro-thrombotic state generated by a high plasma fibrinogen concentration.

For albumin there is a well-characterized redistribution that starts right during the surgical procedure, resulting in a sharp decrease in plasma concentrations [2,4]. There are no major losses of albumin by degradation or loss out of the body that might explain the decrease in concentration, so the initial step is redistribution to the extravascular space. De novo synthesis rates are unaffected or increased. In the hepatectomy group, the synthesis rate for albumin was unchanged throughout the period investigated. Taking into consideration the massive loss of liver tissue following hemihepatectomies, there was indeed an increase in albumin synthesis per tissue unit in the remnant liver. In this study, on postoperative day 1 there was an increase in the albumin synthesis rates in the pancreatectomy group, compared to earlier studies that found the rates to be unaltered on postoperative day 2 [2]. The increase in de novo synthesis of albumin, following pancreatectomies on postoperative day 1 over the preoperative baseline, was as an absolute value around 4 g/day; this was insufficient to influence the plasma albumin concentrations.

It is suggested that albumin synthesis is regulated primarily by the colloid osmotic pressure [32]. Also, inflammation in general has a stimulatory effect on albumin synthesis [33]. In our study albumin synthesis was stimulated on postoperative day 1 but not on postoperative days 3–5, which, under the condition of constant low albumin plasma concentrations, favors inflammation as a stimulatory factor.

It was within our hypothesis that any differences in plasma concentrations of fibrinogen between the two groups of surgical patients studied may be attributable to a difference in liver mass resulting from the surgical procedure. However, there were also some other differences between the two groups regarding fibrinogen utilization, so it is not possible to conclude anything as having been attributable to the differences in liver mass. Still, we intended to have a comparable surgical trauma in the upper abdomen to see what was related to the surgical procedure per se and what was related to the reduction in liver mass.

A predictable postoperative rate of increase in plasma fibrinogen concentration may affect the decision on fibrinogen supplementation. Studies performed in cardiac surgery or cystectomy with massive bleeding show that during the first 24 postoperative hours the plasma fibrinogen concentrations increase at a predictable rate if fibrinogen is not supplemented [34,35]. On postoperative day 1, they reach the same values as preoperatively, regardless whether or not fibrinogen concentrate is administered [34–36], suggesting that the administration of fibrinogen in excess is disposed of by the organism, or slows the synthesis. Here we showed that liver synthesis of fibrinogen following major abdominal surgery is modulated postoperatively to eventually reach the levels of plasma fibrinogen to which the organism is accustomed, which are the preoperative ones. This makes the postoperative plasma fibrinogen concentrations sufficient, even following hemihepatectomies with a low risk of post-hepatectomy liver failure related to low fibrinogen availability.

Studies demonstrate that the regeneration of liver tissue is considerable during the first week after liver resections [37], but there have been no reports until now regarding in vivo synthetic functionality of the new liver tissue in humans. The tendency of albumin synthesis in our patients to increase following hepatectomies towards postoperative days 3–5, when inflammation stimuli were negligible, suggested a good synthetic function of the regenerating liver. The role of fibrinogen in liver regeneration following hepatectomy is discussed increasingly [38] and our study of fibrinogen synthesis in the remnant liver holds significance in this area of research. It is particularly interesting that even a remnant liver can respond with increased fibrinogen synthesis in a situation in which this is the physiologic response. Our study was not designed to elucidate this in quantitative terms, as the size of liver resection was not quantified.

The strengths of our study are the techniques employed to assess the synthesis rate of liver export proteins quantitatively and to put these in relation to functional parameters related to plasma concentrations and to liver function in connection with surgical trauma. All individual values are available to the reader in Figs 1–3. There are also some limitations to our study, such as the relatively small number of subjects that were available, which meant that describing the synthesis trends for patients following extended hemihepatectomies was not possible. However, the size of the increase of the de novo synthesis rate of fibrinogen on postoperative day 1, which was the primary aim of the study, demonstrated that the calculation for statistical power of the study was sufficient. Another notable limitation was the lack of clearance assessments for fibrinogen and albumin.

## Conclusions

In this study, we were able to report patterns for fibrinogen and albumin in terms of plasma concentrations and de novo synthesis perioperatively in conjunction with larger upper abdominal surgery with and without reduction of liver mass. For fibrinogen, there was a dramatic increase in the de novo synthesis rate on postoperative day 1, although plasma concentrations were low following liver resections or unaltered following pancreatic resections. Also, on postoperative days 3–5 the hepatectomy group had a higher de novo synthesis rate of fibrinogen compared to the preoperative values. By the end, the results of these synthesis modifications were, in the hepatectomy group a return to the preoperative plasma fibrinogen concentrations, and in the pancreatectomy group with an intact liver mass, an overshoot in plasma concentrations of fibrinogen. Albumin de novo synthesis was maintained or marginally increased in contrast to the dramatically decreased concentrations. So, despite an over 50% reduction in liver mass after hemihepatectomy, the characteristic response in changes in concentrations and de novo synthesis of fibrinogen and albumin perioperatively remained grossly similar after a major reduction in liver mass as compared to surgery of comparable size. However, the alterations were less pronounced after hepatectomy compared to pancreatectomy, which could possibly have been related to the reduced liver mass.

## Supporting information

S1 Fig Coagulation activation markers. (PDF)

S2 Fig Thromboelastometry. (PDF)
