# Supplementary material for: Fibrinogen and albumin synthesis rates in major upper abdominal surgery
Source: PLoS One. 2022 Oct 27;17(10):e0276775. doi: 10.1371/journal.pone.0276775 (PMC9612515; doi:10.1371/journal.pone.0276775)

Coagulation activation markers. The plasma concentrations of D-dimer, soluble fibrin, thrombin-antithrombin complexes (TAT) and fibrinopeptide B (fpB) in patients undergoing liver surgery (n=9) and pancreas surgery (n=6). The individual values are depicted as black bold points. Levels of statistical significance for the ANOVA (or mixed-effects model analysis within group) are given in the upper right corner. For multiple comparisons, *, **, ***, **** denotes the statistical significance p < 0.05, p < 0.01, p<0.001 and < 0.0001, respectively, of the difference from preoperative values, while # have the same significance from postoperative day 1.

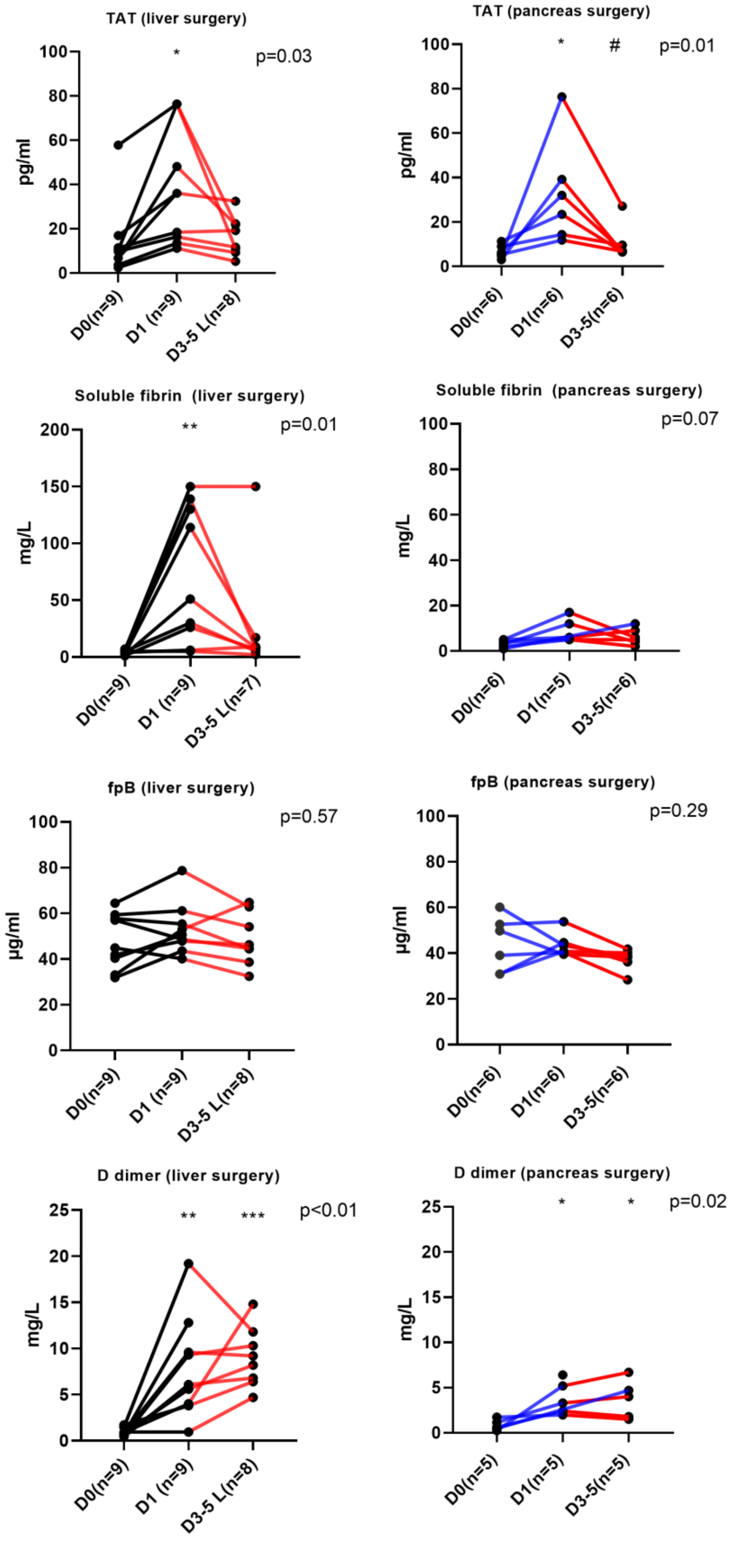

Supplement: S1 Fig — (PDF) [file pone.0276775.s001.pdf]
