# Supplementary material for: Fibrinogen and albumin synthesis rates in major upper abdominal surgery
Source: PLoS One. 2022 Oct 27;17(10):e0276775. doi: 10.1371/journal.pone.0276775 (PMC9612515; doi:10.1371/journal.pone.0276775)

Thromboelastometry. ROTEM parameters (panel A) and fibrinogen fractional synthesis rates and plasma concentrations (panel B) for patients undergoing major liver surgery depicted in red (n=9) and pancreas resection depicted in blue (n=6) (Values are provided as the mean + 0.95 confidence intervals. The grey shaded areas represent the normal ranges). Levels of significance p for mixed-effects model analysis between groups are given in the upper right corner. Levels of statistical significance for the ANOVA (or mixed-effects model analysis within group) are given for each type of surgery (p1 for liver surgery, p2 for pancreas surgery). For multiple comparisons, depicted in red for liver surgery and blue for pancreas surgery, *, **, ***, **** denotes the statistical significance p < 0.05, p < 0.01, p<0.001 and < 0.0001, respectively, of the difference from preoperative values, while # have the same significance from postoperative day 1.

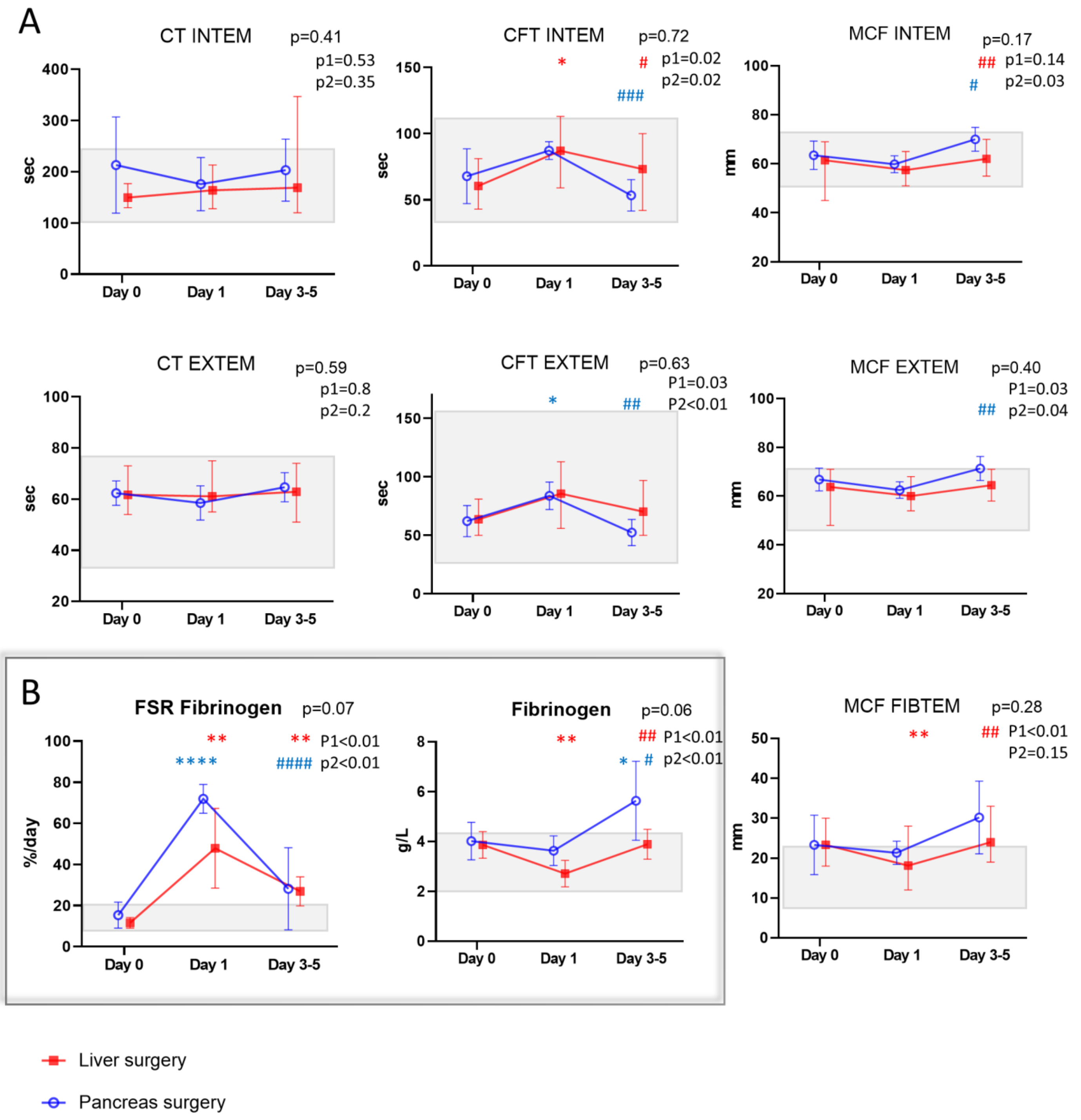

Supplement: S2 Fig — (PDF) [file pone.0276775.s002.pdf]
